# The Comparison of Early Hemodynamic Response to Single-Pulse Transcranial Magnetic Stimulation following Inhibitory or Excitatory Theta Burst Stimulation on Motor Cortex

**DOI:** 10.3390/brainsci13111609

**Published:** 2023-11-20

**Authors:** Corina Gorban, Zhongxing Zhang, Armand Mensen, Ramin Khatami

**Affiliations:** 1 Center for Sleep Medicine, Sleep Research and Epileptology, Clinic Barmelweid, 5017 Barmelweid, Switzerland; corina.gorban@students.unibe.ch (C.G.); mensen19@gmail.com (A.M.); ramin.khatami@barmelweid.ch (R.K.); 2 Department of Neurology, Bern University Hospital, University of Bern, 3012 Bern, Switzerland

**Keywords:** hemodynamics, motor cortex, neuroimaging, spectroscopy, near-infrared, transcranial magnetic stimulation, cerebral blood volume

## Abstract

We present a new study design aiming to enhance the understanding of the mechanism by which continuous theta burst stimulation (cTBS) or intermittent theta burst stimulation (iTBS) paradigms elicit cortical modulation. Using near-infrared spectroscopy (NIRS), we compared the cortical hemodynamics of the previously inhibited (after cTBS) or excited (after iTBS) left primary motor cortex (M1) as elicited by single-pulse TMS (spTMS) in a cross-over design. Mean relative changes in hemodynamics within 6 s of the stimulus were compared using a two-sample *t*-test (*p* < 0.05) and linear mixed model between real and sham stimuli and between stimuli after cTBS and iTBS. Only spTMS after cTBS resulted in a significant increase (*p* = 0.04) in blood volume (BV) compared to baseline. There were no significant changes in other hemodynamic parameters (oxygenated/deoxygenated hemoglobin). spTMS after cTBS induced a larger increase in BV than spTMS after iTBS (*p* = 0.021) and sham stimulus after cTBS (*p* = 0.009). BV showed no significant difference between real and sham stimuli after iTBS (*p* = 0.37). The greater hemodynamic changes suggest increased vasomotor reactivity after cTBS compared to iTBS. In addition, cTBS could decrease lateral inhibition, allowing activation of surrounding areas after cTBS.

## 1. Introduction

Transcranial magnetic stimulation (TMS) is commonly used to study cognitive and executive neurological functions [1]. Usually, single-pulse TMS (spTMS) is used to study brain functions, while repetitive TMS (rTMS) elicits changes in brain activities that persist beyond the stimulation period. Theta burst stimulation (TBS) protocols are specific rTMS paradigms consisting of groups of three 50 Hz pulses repeated at a frequency of 5 Hz [2]. It is known that continuous application of the TBS protocol (cTBS) elicits long-lasting cortical inhibition, whereas intermittent application (iTBS) increases cortical excitability [3,4]. Systematic reviews support the hypothesis that both paradigms effectively affect corticospinal excitability in healthy individuals [5], with the cTBS effect size being stronger overall than iTBS [3,6]. 

The immediate neural response elicited by TMS provides a novel approach to noninvasively study neurovascular coupling mechanisms at the cortical level. However, reliable assessment of the immediate neural response is technically challenging for two major reasons. First, TMS induces a magnetic field that interferes with neuronal coupling, thus limiting the use of EEG [7], SPECT, PET, and fMRI [8,9,10,11]. Second, most of these techniques can only measure neuronal activity at a low temporal resolution, possibly explaining why previous studies averaged multiple TMS stimuli and reported only on the cumulative effect of a train of TMS [9,12,13]. Functional near-infrared spectroscopy (fNIRS) is an optical method that can characterize the local evoked hemodynamics [14,15]. fNIRS provides a unique opportunity to explore cerebral hemodynamics elicited by TMS because the optical signals do not interact with the magnetic or electric fields generated by the TMS coil. fNIRS can measure changes in local intravascular blood volume (BV), oxygenated (HbO_2_), and deoxygenated hemoglobin (HHb) concentrations in the area stimulated by TMS. These provide a multivariable approach to assess various aspects of hemodynamics, such as tissue oxygenation and perfusion. 

To our knowledge, there are no fNIRS data on ipsilateral local hemodynamic changes induced by TBS in the primary motor cortex (M1). We are specifically interested in M1 because stimulation of the motor cortex produces an observable (e.g., thumb movement) and quantifiable change that is measured by electromyography [2,16]. Previous fNIRS studies of hemodynamic changes in the motor cortex induced by rTMS show inconsistent and even contradictory results [17] (see Table 1). These inconsistencies are not restricted to the motor cortex but have been also reported when rTMS is applied to regions other than M1 (see Table 2). Our aim was to compare, for the first time, the evoked hemodynamic changes over M1 after ipsilateral cortical inhibition (cTBS) or facilitation (iTBS) using single-pulse TMS (spTMS). All previous studies have looked at continuous hemodynamic changes (i.e., baseline shift) during or after rTMS. Since our interest is the hemodynamic changes elicited by spTMS, we focused on the responsiveness of the previously excited or inhibited cortex, not on baseline hemodynamic shifts elicited by rTMS itself. This experimental design should enable a clearer comprehension of the discrepancies previously documented in the literature. We believe that any additional findings on the effects of TMS could help to better understand its mechanism and expand its range of applications. Based on the results of previous TBS studies [3,6] demonstrating stronger effects of cTBS on neuronal activities, we hypothesized that spTMS after cTBS modulation could induce a more pronounced cortical hemodynamic effect compared to the one after iTBS modulation.

## 2. Materials and Methods

### 2.1. Subjects

Data were collected from 10 right-handed participants (mean age 40 years, range: 18–60 years), recruited by local advertisement. Exclusion criteria were a history of epilepsy, a history of brain injury, the presence of other major medical illnesses, neurological or psychiatric clinical history, intake of medication during the study, and history of drug intake. All selected participants were healthy males with a bald head and no dark skin, in order to achieve better fNIRS signals. The experimental procedure was approved by the Ethics Committee Northwest/Central Switzerland (EKNZ, Reference-Nr. 2013/078) and was performed in accordance with relevant guidelines and regulations. All participants gave their written informed consent for the experiment and were paid for participation.

### 2.2. Procedure

Eight subjects participated in both the cTBS and iTBS sessions, at least one week apart, in randomized order. One subject underwent cTBS only and one subject underwent iTBS only. The fNIRS measurement was performed continuously before TBS, during TBS, and after TBS modulation up to a total of 1 h. Participants reported no adverse effects (e.g., headache and dizziness) after any of the TBS sessions.

### 2.3. spTMS and TBS

Magnetic stimulation was performed using the MagPro X100 with a water-cooled figure-of-eight coil, which was always held with the handle facing backwards and at an angle of approximately 45° to the midline [29]. A neuro-navigation system was used to precisely place the TMS coil over the hand region of the motor cortex in the left hemisphere (M1) throughout the session using a standardized MRI image adapted to the head shape. A motor-evoked potentials (MEPs) recorder with surface electrodes assessed the motor responses of the right first dorsal interosseous muscle (FDI) to single biphasic TMS pulses over the corresponding left M1 for localization purposes. The optimal coil position with the most elicited MEPs, confirmed by the visualized movement of the right index finger, was defined as the stimulation point. It was marked with a fine pencil on the scalp. The fNIRS probe (i.e., a rubber patch) was placed over the stimulation point. A photo of the experimental setup was given in the Supplementary Materials.

**Figure 1:**
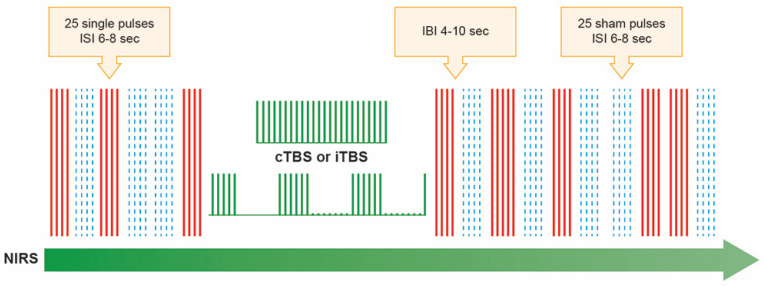
Illustration of the stimulation paradigms used. Using near-infrared spectroscopy (NIRS), the hemodynamic changes were measured when TMS blocks of single pulses, both real (25 pulses at 100% of resting motor threshold, marked as solid red lines) and sham (25 pulses that produce a clicking sound that was the same as an active TMS pulse but without actual stimulation of the brain, which were marked in blue dash lines), were applied across M1 before (3 blocks of real and 3 blocks of sham stimulus, which were randomized) and after (5 blocks of real and 5 blocks of sham stimulus, which were randomized) transcranial magnetic theta burst stimulation at 80% of active motor threshold. The theta burst stimulation pattern (TBS), in which 3 pulses of stimulation were given at 50 Hz, repeated every 200 ms (5Hz). In the continuous theta burst stimulation paradigm (cTBS), a 40 s train of uninterrupted TBS was given (600 pulses). In the intermittent theta burst stimulation pattern (iTBS), a 2 s train of TBS was repeated every 10 s for a total of 190 s (600 pulses). Randomly determined inter-block interval (IBI) was 4–10 s. The randomized inter-stimulus interval (IBI) was 6–10 s. Therefore, one session lasted about 40 to 58 min, depending on the randomized ISI, IBI, and the TBS paradigm (i.e., cTBS or iTBS).

The TMS protocol is illustrated in Figure 1. It consisted of 3 blocks (random interval between blocks 6–10 s) of single pulses at 100% of resting motor threshold (RMT) before TBS and 5 blocks of single pulses at 100% of RMT after TBS. Each block consisted of 25 magnetic pulses (randomized inter-stimulus interval, ISI: 6–8 s) and 25 sham pulses (ISI: 6–8 s) in a randomized order. The sham pulse produces a clicking sound that is the same as an active TMS pulse but without actual stimulation of the brain. The RMT was considered the lowest magnetic stimulus intensity required to produce an MEP of at least 50 µV on at least 3 of 5 trials from the relaxed right FDI. Higher RMT values indicate low cortical excitability, while lower RMT values indicate high cortical excitability. As cTBS reduces cortical excitability, a higher intensity (i.e., 100% rMT) was considered more appropriate for stimulation. During sham stimulation, the same active coil was tilted 90° and only the handle remained in contact with the fNIRS probe [36] so that participants were blinded to the real and sham stimulation.

The iTBS and cTBS protocols were constructed analogously to the classical excitation and inhibition protocols of Huang et al. [2]. For TBS, the stimulation intensity was set at 80% of the active motor threshold (AMT), which is considered the lowest TMS intensity required to elicit an MEP during a mild tonic contraction of the thumb (approximately 20% of maximal force) while holding a cylindrical object and which is better tolerated when applied at high frequency.

### 2.4. Frequency-Domain Multi-Distance (FDMD) Near-Infrared Spectroscopy (NIRS)

The FDMD-NIRS measurements were performed using the only commercially available system from ISS (Imagent, ISS, Champaign IL, USA). The principle of FDMD-NIRS was already well established [37,38]. In the Imagent system, the light sources (8 laser diodes, 4 with a wavelength of 690 nm and 4 with a wavelength of 830 nm, i.e., 4 coupled sources) are modulated at 110 MHz, and the light can penetrate the tissue under examination at a depth of about 3–4 cm from the scalp. TMS activates the M1 at a depth of 1.5–2.1 cm [39], i.e., at the level of deep cortical layers or at the border between gray and white matter. The light backscattered from the tissue can be picked up by a 3 mm diameter optical fiber bundle coupled to a photomultiplier detector. To allow measurement over multiple distances, the four coupled light sources were aligned and placed at 2 cm, 2.5 cm, 3 cm, and 3.5 cm from the detecting optical fiber bundle. The light sources and the distal end of the bundle were sealed with a 4 mm thick rubber patch. The measured light intensity ($I_{\mathrm{DC}}$), modulation amplitude ($I_{\mathrm{AC}}$), and phase from different distances vary linearly (linearity was checked using the R^2^ of the fitted linear regression model). Thus, subjecting the measured values $I_{\mathrm{DC}}$, $I_{\mathrm{AC}}$, and phase to linear regression, the following equations are obtained [37,38,40]:

(1) $$ln(r^{2}I_{\mathrm{AC}})=rS_{\mathrm{AC}}+C_{\mathrm{AC}}$$

(2) $$ln(r^{2}I_{\mathrm{DC}})=rS_{\mathrm{DC}}+C_{\mathrm{DC}}$$

(3) phase=rSphase+Cphase

where r is the known source-detector distance; $S_{\mathrm{AC}}$, $S_{\mathrm{DC}}$, and $S_{\mathrm{phase}}$ are the slopes; and $C_{\mathrm{AC}}$, $C_{\mathrm{DC}}$, and $C_{\mathrm{phase}}$ are the intercepts. Cerebral hemodynamic parameters were calculated using the slopes; that is, to combine two of these three slopes, we can estimate the absorption and reduced scattering coefficients of the measured tissue and then further calculate HbO_2_ and HHb. Total hemoglobin reflecting BV changes was then calculated as the sum of HbO_2_ and HHb. We chose $S_{\mathrm{AC}}$ and $S_{\mathrm{phase}}$, considering that $I_{\mathrm{AC}}$ and phase are less contaminated by environmental light. The sample rate of our FDMD-NIRS recording was set as 10.4 Hz. Before starting each measurement, the NIRS device was calibrated on optical phantom blocks.

The raw hemodynamic fNIRS signals were first subjected to a bandpass filter using a Hanning window with a cutoff frequency of 0.02 Hz to remove baseline deviations and 0.7 Hz to filter the physiological noise of the heartbeat [41,42,43]. The reliability of the FDMD-fNIRS measurement depends on the linearity of the raw optical signals as a function of distance, i.e., the linear dependence R^2^ of Equations (1) and (3) should be very close to 1. We then checked the R^2^ of the regression fit and the *p*-values for the linearly fitted absorption and reduced scattering coefficients. The data were discarded if the R^2^ was smaller than 0.97 in either modulation amplitude or phase shift [41,42,43]. Some fNIRS changes induced by spTMS events were excluded from further analysis if more than 1/3 of the raw optical data were discarded after spTMS. fNIRS data with clear motion artifacts (i.e., head, body, hands, or thumb movements of the subjects) were also discarded. The remaining data were segmented (i.e., data fragments after each spTMS). In each data fragment, the time of spTMS was treated as baseline and subtracted from the post-stimulation recordings. The relative NIRS changes were then normalized to the baseline to allow a valid group-level comparison. The same normalization was performed for cerebral HbO_2_, HHb, and BV.

### 2.5. Statistical Analysis

Normalized relative changes in NIRS signals between the true and sham stimuli, between signals after cTBS and iTBS, and between signals before and after modulation were subjected to a two-sample Welch *t*-test (*p* < 0.05). For those subjects who finished both cTBS and iTBS protocols, we used a linear mixed model (LMM) with a random intercept by subject to further compare the changes in NIRS signals between baseline without stimuli, after cTBS and iTBS. All signal processing was performed in MATLAB 2017b. All statistical analyses and modeling were performed using R (version 3.5.3). The two-sample Welch *t*-test was performed using the R package stats and LMM was completed with the R package *nlme*.

## 3. Results

Six recordings (i.e., subjects) with cTBS and seven recordings with iTBS were validated for analysis. The other data of the subjects were excluded from analysis due to poor fNIRS signal quality.

The total number of validated (i.e., without influences of motion artifacts and with an R^2^ of the FDMD-NIRS data greater than 0.97) is presented in Table 3. The total numbers of real and sham stimuli after cTBS were 684 and 447, respectively. These numbers were 702 and 647 for iTBS, respectively. The total numbers of real stimulus before cTBS and iTBS (i.e., pre-TBS), acquired from all the subjects, were 395 and 402, respectively. These numbers were 256 and 354 for sham stimulus before cTBS and iTBS, respectively.

**Figure 2:**
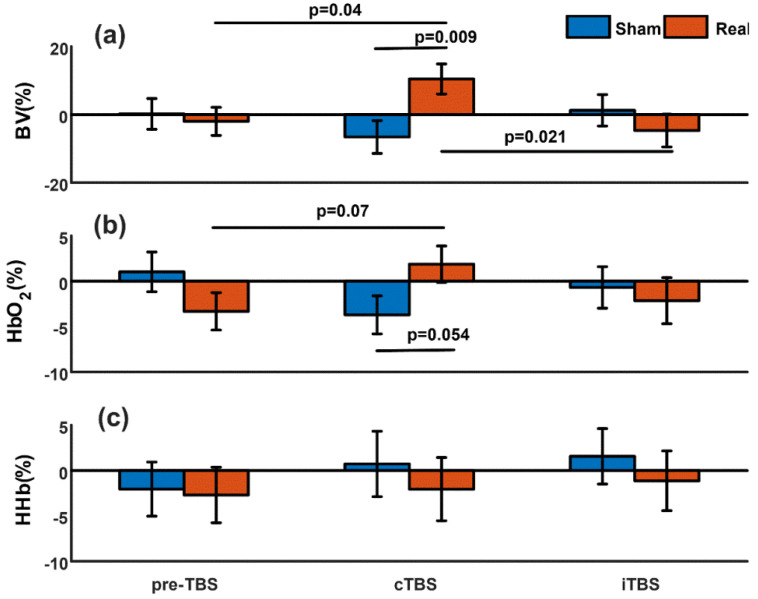
Summary of the evoked changes in NIRS measurements. Mean normalized relative changes in cerebral blood volume (BV) (**a**), oxygenated hemoglobin (HbO_2_) (**b**), and deoxygenated hemoglobin (HHb) (**c**) elicited by real and sham single-pulse TMS stimulation before theta burst stimulation (preTBS), after continuous theta burst stimulation (cTBS), and after intermittent theta burst stimulation (iTBS). The error bar represents standard error. The *p*-values above the horizontal lines indicate that the values at the two ends of the lines are significantly different (*p* < 0.05) or tend to be significantly different (*p* < 0.1).

No significant differences were found in NIRS signals evoked by real spTMS stimulus before cTBS and before iTBS (BV: *p* = 0.31, HbO_2_: *p* = 0.33, HHb: *p* = 0.57) or by sham spTMS stimulus before cTBS and before iTBS (BV: *p* = 0.45, HbO_2_: *p* = 0.78, HHb: *p* = 0.31), indicating a similar NIRS baseline before cTBS and iTBS modulations in our subjects. Therefore, we combined the stimulus at the baseline before both TBS modulations, resulting in a total of 797 real and 610 sham stimuli before TBS. Figure 2 summarizes the changing trends of NIRS signals in our experiment.

### 3.1. Evoked Blood Volume Changes: Real vs. Sham

There was no significant difference (*p* = 0.72) between the real and sham spTMS stimulus in the baseline period before TBS modulations. After cTBS, real spTMS induced significantly greater changes in BV compared to sham spTMS (*p* = 0.009, Figure 2a). In contrast, after iTBS, there was no significant difference in BV changes between real and sham spTMS (*p* = 0.37).

### 3.2. Evoked Blood Volume Changes: After cTBS vs. after iTBS

Real spTMS after cTBS induced significantly increased cerebral BV compared to real spTMS after iTBS (*p* = 0.021), while there was no significant difference between evoked BV induced by sham spTMS after cTBS and iTBS (*p* = 0.24).

### 3.3. Evoked Blood Volume Changes: After TBS vs. Baseline before TBS

Comparing the stimuli before and after cTBS/iTBS, we found that only the evoked BV induced by real spTMS after cTBS was significantly higher than that induced by real spTMS at baseline before TBS modulations (*p* = 0.04).

### 3.4. Evoked Oxygenated Hemoglobin (HbO_2_)

At baseline (pre-TBS), evoked HbO_2_ changes were similar between real and sham spTMS (*p* = 0.15). HbO_2_ changes triggered by real spTMS tended to be greater than those elicited by sham spTMS after cTBS (*p* = 0.054, Figure 2b). The HbO_2_ increase also tended to be significant when we compared the changes in HbO_2_ triggered by real spTMS before TBS modulations and after cTBS (*p* = 0.07). No significant difference was found between real and sham spTMS after iTBS (*p* = 0.67) or between the real spTMS after cTBS and iTBS (*p* = 0.21).

### 3.5. Deoxygenated Hemoglobin (HHb)

We did not find any significant differences in the evoked HHb changes between real and sham spTMS at baseline before TBS (*p* = 0.88), after cTBS (*p* = 0.58), or after iTBS (*p* = 0.55).

### 3.6. Linear Mixed Model Analysis

The relatively small number of subjects may cause false-positive results in the *t*-test in our aforementioned analyses. We therefore further run LMM with subject as random intercept using data from five subjects who successfully finished both cTBS and iTBS paradigms and checked whether LMM analysis can confirm the results of *t*-tests. The number of five subjects was still small, but their within-subject data from a crossover design provided a more efficient comparison compared to the *t*-test. We only applied LMMs on the changes in BV and HbO_2_, because in Figure 2, we found significant or almost significant differences in these two parameters between pre-TBS, cTBS, and iTBS. In total, valid NIRS data of 654 real spTMS stimuli at baseline before TBS, 576 real spTMS stimuli after cTBS, and 579 real spTMS stimuli after iTBS, measured from these five subjects, were used to fit the LMM. Similarly, the numbers of sham stimuli at baseline before TBS, after cTBS, and after iTBS were 521, 356, and 561, respectively.

LMMs confirmed the results shown in Figure 2a, i.e., the BV triggered by real spTMS after cTBS was significantly higher than the ones pre-TBS (mean difference = 13.86%, standard error (SE) = 6.60, *p* = 0.036), while there is no significant difference between BV after iTBS and pre-TBS (mean difference = −1.36%, SE = 6.59, *p* = 0.84). The changes in BV are also significantly higher after cTBS compared to after iTBS (mean difference = 15.22%, SE = 6.80, *p* = 0.025). There were no significant differences in the BV triggered by sham stimuli between pre-TBS and after cTBS (mean difference = 2.10%, SE = 7.13, *p* = 0.77), between pre-TBS and after iTBS (mean difference = 1.96%, SE = 6.32, *p* = 0.76), or between after cTBS and after iTBS (mean difference = −0.13%, SE = 7.08, *p* = 0.98).

Similarly, LMMs also confirmed the results shown in Figure 2b. The HbO_2_ triggered by real spTMS after cTBS showed a trend to be significantly higher than the ones pre-TBS (mean difference = 6.19%, SE = 3.21, *p* = 0.054), but there was no significant difference between pre-TBS and after iTBS (mean difference = -3.61%, SE = 3.21, *p* = 0.26). There was no significant difference between real spTMS stimuli after cTBS and iTBS (mean difference = 2.58%, SE = 3.31, *p* = 0.44) either. No significant differences were found in HbO_2_ triggered by sham stimuli between pre-TBS and after cTBS (mean difference = 2.73%, SE = 3.29, *p* = 0.41), between pre-TBS and after iTBS (mean difference= 3.49%, SE = 2.91, *p* = 0.23), or between after cTBS and after iTBS (mean difference = 0.76%, SE = 3.24, *p* = 0.81).

## 4. Discussion

As a primary result, our study showed a significant increase in BV after cTBS, while spTMS failed to induce significant BV changes after iTBS. spTMS after both cTBS and iTBS did not cause significant changes in HbO_2_ or HHb. HbO_2_ tended to increase following spTMS after cTBS but did not reach statistical significance.

We used a rigorous experimental design with sham and real stimulation to control for potential confounding factors such as systematic hemodynamic effects and noise from the TMS device. More importantly, by pooling the stimuli at baseline before the TBS modulations, we controlled for baseline shifts induced by the initial spTMS. It is noteworthy that our novel experimental approach could make a valuable contribution to various aspects of neurovascular coupling and vasomotor activity and may help to understand the conflicting results in the literature.

Existing studies provide different results on the changes in hemodynamics induced by rTMS. These changes have been shown to be highly dependent on the type of rTMS, the intensity and location of the stimulations, and the NIRS measurement techniques, making it difficult to compare the existing results in the literature (see Table 1 and Table 2). In addition, the different NIRS techniques, i.e., continuous wave (CW) vs. FDMD, may also contribute to the inconsistent results. Hada et al. [21] and Näsi et al. [22] reported that BV measured with CW-NIRS in M1 changed in a similar manner regardless of whether rTMS inhibited or activated neuronal excitation. Näsi et al. found the same pattern of cerebral BV changes in three CW-NIRS measurements (short, medium, and long source-detector channels), which was evident even when the same rTMS paradigms were applied to the subjects’ shoulder instead of M1. Näsi et al. also showed that the changes in heart rate and photoplethysmograph amplitude correlated with the changes in BV measured by CW-NIRS, suggesting that systemic and peripheral hemodynamics may contribute more to the CW-NIRS signals. In contrast, FDMD-NIRS is an advanced technique that is more sensitive to cortical hemodynamics [44,45]. The FDMD-NIRS measurements in the left dorsolateral prefrontal cortex show that BV changes differently during inhibitory and excitatory rTMS [30], which may be more plausible to reflect different cortical activity induced by different rTMS paradigms. Our results of different changes in BV between real and sham rTMS after cTBS suggest that our FDMD-NIRS captured the hemodynamic changes induced by local neuronal activity rather than global blood flow effects; otherwise, BV would have changed similarly. The methodological issue of NIRS is crucial and must be considered in any TMS-NIRS study. In the case of CW-NIRS measurement, a larger portion of the signal could originate from the extracranial or global circulation rather than the cortex, leading to misinterpretation of cerebral neural activity.

We also used the specially designed thin NIRS patch probes (4 mm thick) directly on the bald scalp to avoid attenuation of light intensity by hair absorption as much as possible. Otherwise, a low signal-to-noise ratio and inhomogeneity of the extracerebral signal may occur [46], violating the assumption of homogeneity of the FDMD-NIRS algorithm [38]. Finally, our results with no difference between sham and real spTMS during the period before the TBS modulations (baseline) suggest that a potential baseline shift of BV was unlikely to be co-induced. The spTMS stimuli at a minimal 6 s interval did not cause a baseline hemodynamic shift before TBS, and only spTMS after TBS caused hemodynamic changes. This confirms the validity of our experimental design, so the effects of real and sham spTMS before iTBS and cTBS were pooled for further comparison.

Our main result of stronger evoked cortical hemodynamics after cortical inhibition compared to cortical excitation is consistent with previous studies that demonstrated stronger effects of cTBS on neural activity than iTBS [3,6]. We consider the following possible mechanisms to explain why cerebral perfusion and oxygenation elicited by spTMS increased after cTBS but not after iTBS:

1. cTBS increases cerebral vasomotor reactivity (VMR)

VMR is the ability of arterioles to vasodilate in response to external stimuli. Vernieri et al. indicated that high-frequency (17 Hz) rTMS applied to the M1 area can produce a significant bilateral decrease in cerebral VMR in healthy subjects and stroke patients [47]. Another study by the same group showed that inhibitory rTMS applied to M1 at 1 Hz produced a bilateral, long-lasting (at least 5 h) increase in cerebral VMR [48]. A plausible interpretation of our results is that cTBS increases the VMR of targeted cortical vessels, increasing the potential for vasodilation and exceeding the vasoconstriction expected from spTMS [22]. Consequently, the net vascular response can be viewed as the result of the simultaneous effects of vasodilators and vasoconstrictors, with the balance in favor of vasodilation [49]. Our finding of significantly increased BV and HbO_2_ at relatively constant HHb triggered by real spTMS after cTBS is a typical pattern for vasodilatation. Moreover, the observed greater increase in BV than in HbO_2_ after cTBS, a combination of stimulus-induced hemodynamic changes consistent with the positive fMRI-BOLD signal, is known to be elicited by vasodilators [49]. Because VMR is determined by the extent of stimulus-induced vasodilation, we argue that our results are consistent with the concept of increased VMR.

2. cTBS reduces lateral inhibition, allowing the activation of the surrounding area

Lateral inhibition (or surrounding inhibition) is a ubiquitous process in the central nervous system in which excited neurons inhibit the action of neighboring neurons so that central signals are promoted, and eccentric signals are inhibited to enhance the contrast between them [50]. This mechanism allows neurons to discriminate how much action potential is generated within the stimulation point and in its vicinity. It has previously been postulated that cTBS may reduce the efficacy of transmission through the synaptic connections recruited at the stimulation point [2], thereby reducing lateral inhibition (i.e., neighboring neurons may respond more actively to stimuli). Consequently, spTMS after CTBS generates action potentials not only in target neurons but also in neighboring neurons that should normally be inhibited. This results in spatial and temporal spread of cortical activity and reduced motor evoked potential. We argue that action potential propagation could also trigger energy-consuming processes in surrounding neurons, leading to increased BV around the stimulation area via neurovascular coupling. This hypothesis is also supported by the fact that our FDMD-NIRS measures a large area around the stimulation point (~1 to 10 cm^3^ tissue volume [51]).

Leodori et al. associate the mechanism of lateral inhibition with short-interval intracortical inhibition (SICI) [52]. SICI is a complex mechanism involving low-threshold inhibitory interneurons which has been demonstrated in studies using paired-pulse TMS and occurs at very short inter-stimulus intervals of 1–5 ms [53]. According to Leodori et al., the same inhibitory interneurons may be the final effector of both SICI and lateral inhibition. Our assumption that cTBS causes a reduction in lateral inhibition leading to a lateral spread of cortical activation is also supported by Huang et al. [2], who postulate that one of the mechanisms of cTBS is the reduction in SICI.

The present study has several limitations. First, our paradigm does not allow us to quantify the cumulative effect of TBS. Our goal is to study the immediate effect; therefore, we must administer real and sham spTMS randomly after TBS, which makes it impossible to assess baseline hemodynamic changes. Second, we have a limited number of subjects because we measured subjects with bald heads and had to use FDMD-NIRS with a thin patch probe. In a recent study, Curtin et al. systematically reviewed 14 published studies including several studies listed in Table 1 in our study that measured M1 during TMS stimulations using fNIRS [17]. Only 6 studies measured more than 10 subjects (i.e., 3 studies included 11 subjects each, one study measured 12 subjects, and one study measured 15 subjects). In 5 studies, the number of subjects was between 6 and 8, which was similar to our study. It is reasonable to assume that the relatively small number of subjects in those previous studies is due to the difficulty of measuring motor cortex using CW-NIRS; probably because only optodes can measure the brain area covered by hair but they are much higher than patch probes. Thus, optodes can attenuate the TMS effect on the cortex. We were well aware of the limitation of a small number of subjects; thus, we did both *t*-test and LMM analysis in a crossover design to confirm the same results, which could minimize the potential false-positive error in our analyses. However, we suggest that our results need to be reproduced in future studies with a larger population. Third, the relatively short ISI prevents us from testing whether later deactivation can be found in the FDMD-NIRS measurement, which would provide further insight into the physiology of TBS. Previous studies reported later deactivation after spTMS, i.e., decreased BV occurs 9–12 s after stimulation [19,20]. Our paradigm limits the study of later hemodynamics because our ISI is 6–8 s, so we only characterize evoked hemodynamics within 6 s after spTMS. If the phenomena of later deactivation are real, this could explain the results showing no significant changes between real and sham stimuli before TBS, because our NIRS signals could be a mixture of early activation and later deactivation.

## 5. Conclusions

Our main finding of stronger evoked cortical hemodynamics after cortical inhibition compared to cortical excitation is consistent with previous studies showing stronger effects of cTBS on neuronal activity than iTBS [3,6]. We suggest that increased VMR and/or reduced lateral neuronal inhibition may be the underlying mechanisms of cTBS. TBS protocols are increasingly used for therapeutic purposes, such as in patients with stroke [54,55,56,57] or psychiatric disorders [58,59,60,61]. Therefore, research in this area should continue to be of interest to researchers and clinicians as it may lead to more effective treatments or interventions.

## Figures and Tables

**Table 1:** Synopsis of NIRS-TMS studies on left motor cortex (M1).

TMS Frequency Type	TMS Protocol	TMS Intensity	NIRS Methodology	Results	Reference
High-Frequency rTMS	3 trains of 120 s of QPS-5 (4 pulses ISI 5 ms) or QPS-50 (ISI 50 ms), ITI 5 s	90% AMT	20 s after each train onset until its end (avg), ipsilateral	QPS-5 trains: oxy-Hb ↑	Groiss et al., 2013 [18]
	20 bursts of QPS-5/QPS-50, IBI 55 s	90% AMT	5 s to 20 s after each burst (avg), ipsilateral	QPS-5 burst: oxy-Hb ↓	Groiss et al., 2013 [18]
Single-Pulse TMS	20 spTMS at random ISI 24–26 s, repeated 2–3 times	100% AMT	5 s pre and 20 s post each spTMS, ipsilateral	Hb -	Mochizuki et al., 2006 [19]
	20 spTMS at random ISI 24–26 s, repeated 2–3 times	120% AMT	5 s pre and 20 s post each spTMS, ipsilateral	deoxy-Hb ↓ tHb ↓ oxy-Hb -	Mochizuki et al., 2006 [19]
	20 spTMS at random ISI 24–26 s, repeated 2–3 times	140% AMT	5 s pre and 20 s post each spTMS, ipsilateral	deoxy-Hb ↓ tHb ↓ oxy-Hb -	Mochizuki et al., 2006 [19]
	spTMS	100–140% AMT	3 s pre and 13.5 s post each spTMS, ipsilateral	oxy-Hb ↑ post-spTMS, peak at 3–6 s	Furubayaschi et al., 2013 [20]
Low-Frequency rTMS	1 train of 10 pulses at 0.5 Hz	120% RMT	10 s before, during and 170 s after rTMS	oxy-Hb ↓ immediately after initiation of rTMS	Hada et al., 2006 [21]
	1 train of 10 pulses at 0.5 Hz	80% RMT	10 s before, during and 170 s after rTMS	oxy-Hb ↓ 5 s after initiation of rTMS	Hada et al., 2006 [21]
	25 trains of 8 s at 0.5 Hz rTMS	70% RMT	during rTMS and 20 s after rTMS, ipsilateral	during rTMS: oxy-Hb ↓ post-rTMS: oxy-Hb ↑	Näsi et al., 2011 [22]
	15 trains of 10s at 1 HZ (ITI 80 s)	120% AMT	1 min pre-, during, 1 min post-rTMS, bilateral	during rTMS: oxy-Hb ↓ bilateral and more contralateral	Kozel et al., 2009 [23]
	25 trains of 8 s at 1 Hz rTMS	70% RMT	during rTMS and 20 s after rTMS, ipsilateral	during rTMS: oxy-Hb ↓ post-rTMS: oxy-Hb ↑	Näsi et al., 2011 [22]
	1 train of 20 min at 1 Hz rTMS	95% RMT	during and 4 min post-rTMS, ipsilateral	during rTMS: oxy-Hb ↑, CBF ↑ Plateau at ca. 15 min	Mesquita et al., 2013 [24]
	500 pulses at 1 Hz rTMS	90–120% RMT	during motor task before and after rTMS, bilateral	Hb -	Furukawa et al., 2021 [25]
	1 train of 19 min at 1 Hz rTMS	90% AMT	19 min during and 20 min post-rTMS, contralateral	oxy-Hb ↑ continuously and was maintained for 20 min after	Park et al., 2017 [26]
	1 train of 10 pulses at 2 Hz	120% RMT	10 s before, during and 170 s after rTMS	oxy-Hb ↓ immediately after initiation of rTMS	Hada et al., 2006 [21]
	1 train of 10 pulses at 2 Hz	80% RMT	10 s before, during and 170 s after rTMS	oxy-Hb ↓ 5 s after initiation of rTMS	Hada et al., 2006 [21]
	25 trains of 8 s at 2 Hz rTMS	70% RMT	during rTMS and 20 s after rTMS, ipsilateral	during rTMS: oxy-Hb ↓ post-rTMS: oxy-Hb ↑	Näsi et al., 2011 [22]

ISI, inter-stimulus interval; ITI, inter-train interval; QPS-5/-50, quadri-pulse stimulation with ISI 5 or 50 ms; rTMS, repetitive TMS; spTMS, single-pulse TMS; AMT, active motor threshold; RMT, resting motor threshold; ↑, increase; ↓, decrease.

**Table 2:** Synopsis of NIRS-TMS studies on the dorsolateral prefrontal or prefrontal cortex (DLPFC/PFC).

TMS Frequency Type	TMS Protocol	TMS Intensity	NIRS Methodology	Results	Reference
Single-Pulse TMS	1 train of 2 or 4 spTMS (ISI 5 s)	130% AMT	5 s period pre-, 15 s and 25 s period post-spTMS, ipsilateral	oxy-Hb ↓	Thomson et al., 2012 [27]
	30 spTMS (ISI 25 s)	130% RMT	5 s interval at around 10 s post-rTMS, ipsilateral	oxy-Hb ↓	Thomson et al., 2013 [28]
Low-Frequency rTMS	30 trains of 20 s at 1 Hz rTMS (ITI 40 s)	120% RMT	10 s interval at around 20 s post-rTMS, ipsilateral	oxy-Hb ↑	Thomson et al., 2013 [28]
	2 trains of 10 min at 1 Hz rTMS, ITI 20 min	80% RMT	during rTMS, split in 5 blocks of 2 min, avg, ipsilateral	oxy-Hb ↓ during rTMS, returning to baseline in 5 min	Thomson et al., 2012 [29]
	2 trains of 10 min at 1 Hz rTMS, ITI 20 min	120% RMT	during rTMS, split in 5 blocks of 2 min, avg, ipsilateral	oxy-Hb ↓ during rTMS, returning to baseline in 10 min	Thomson et al., 2012 [29]
	20 trains of 5 s at 1 Hz rTMS, ITI 25 s	110% RMT	during rTMS and 20 s after rTMS, ipsilateral	during rTMS: oxy-Hb ↓ post-rTMS: oxy-Hb ↑	Cao et al., 2013 [30]
	1 train of 60 s at 1 Hz rTMS	50% RMT	20 s before, 60s during and 120 s after rTMS, ipsilateral	during rTMS: oxy-Hb ↓ post-rTMS: oxy-Hb ↑	Hanaoka et al., 2007 [31]
	1 train of 60 s at 1 Hz rTMS	58–100% RMT	during and 120 s post-rTMS, contralateral	during rTMS: oxy-Hb ↓ post-rTMS: oxy-Hb ↑	Aoyama et al., 2009 [32]
High-Frequency rTMS	20 trains of 5 s at 5 Hz rTMS, ITI 25 s	110% RMT	during rTMS and 20 s after rTMS, ipsilateral	during rTMS and post-rTMS: oxy-Hb ↑	Cao et al., 2013 [30]
	20 SICI pulses (ISI 2 ms), 20 ICF pulses (ISI 15 ms), 20 spTMS (ISI 25 s)	70% RMT	pre- and 25 s post-stimulus (averaged), ipsilateral	oxy-Hb ↓	Thomson et al., 2011 [33]
	15 trains of 5 s at 10 Hz rTMS, ITI 55 s (20 min)	110% RMT	oxy-Hb 4 h after rTMS, during task	oxy-Hb ↓ during task, 4 h after rTMS	Sutoh et al., 2016 [34]
TBS	classic cTBS	80% RMT	pre-cTBS and post-cTBS, contralateral	oxy-Hb ↓ (contralateral)	Tupak et al., 2013 [35]

ICF, intracortical facilitation; ISI, inter-stimulus interval; ITI, inter-train interval; rTMS, repetitive TMS; SICI, short-interval intracortical inhibition; spTMS, single-pulse TMS; AMT, active motor threshold; RMT, resting motor threshold; ↑, increase; ↓, decrease.

**Table 3:** The total number of validated real and sham stimuli before and after cTBS and iTBS.

	Nr. of Real Stimuli		Nr. of Sham Stimuli	
Before cTBS	395	Total 797	256	Total 610
Before iTBS	402		354	
After cTBS	684		447	
After iTBS	702		647	

## Data Availability

The datasets generated and analyzed during the current study are available from the corresponding author upon reasonable request.

## Supplementary Materials

The following supporting information can be downloaded at: https://www.mdpi.com/article/10.3390/brainsci13111609/s1, Figure S1: The set-up of experiment. Magnetic stimulation was performed using the MagPro X100 with a water-cooled figure-of-eight coil.
