# Supplementary material for: The Comparison of Early Hemodynamic Response to Single-Pulse Transcranial Magnetic Stimulation following Inhibitory or Excitatory Theta Burst Stimulation on Motor Cortex
Source: Brain Sci. 2023 Nov 20;13(11):1609. doi: 10.3390/brainsci13111609 (PMC10670137; doi:10.3390/brainsci13111609)
Supplement: Supplementary file 1 [file brainsci-13-01609-s001.zip › brainsci-2686891-supplementary.pdf]

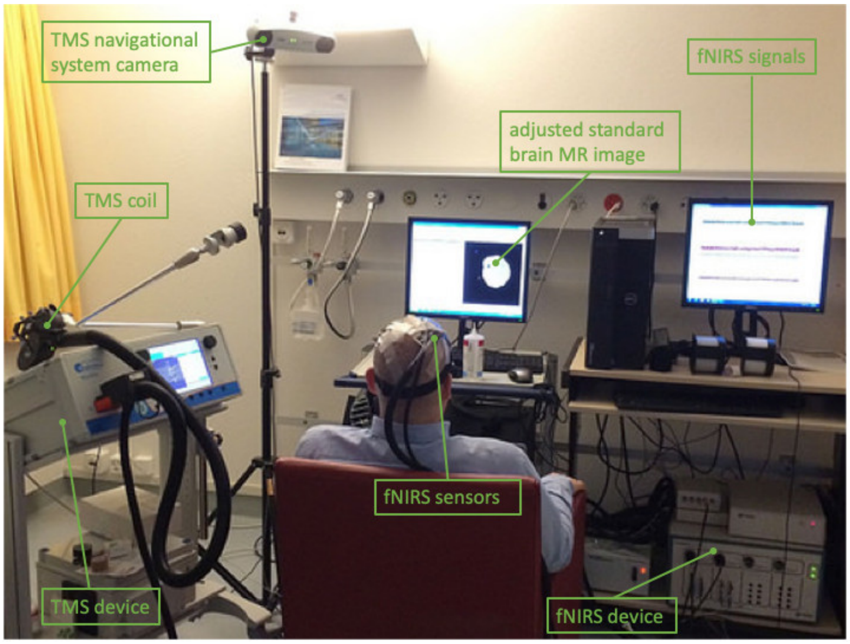

Figure S1. The set-up of experiment. Magnetic stimulation was performed using the MagPro X100 with a water-cooled figure-of-eight coil. A neuro-navigation system was used to precisely place the TMS coil over the hand region of the motor cortex in the left hemisphere (M1) throughout the session using a standardized MRI image adapted to the head shape. The optimal coil position with the most elicited motor evoked potentials, confirmed by visualized movement of the right index finger, was defined as the stimulation point. The fNIRS probe (i.e., a rubber patch) was placed over the stimulation point.
